# Mapping the Kinase Inhibitor Landscape in Canine Mammary Carcinoma: Current Status and Future Opportunities

**DOI:** 10.3390/ani16020232

**Published:** 2026-01-13

**Authors:** Małgorzata Chmielewska-Krzesińska

**Affiliations:** Laboratory of Genomics and Transcriptomics, Department of Pathophysiology, Forensic Veterinary Medicine and Administration, Faculty of Veterinary Medicine, University of Warmia and Mazury in Olsztyn, 10-719 Olsztyn, Poland; malgorzata.chmielewska@uwm.edu.pl

**Keywords:** canine mammary carcinoma, human breast cancer, kinase inhibitors, targeted therapy, translational oncology, PI3K/AKT/mTOR pathway, CDK4/6-RB pathway, HER2 pathway

## Abstract

**Background**: Canine mammary carcinoma (CMC) is the most common malignant tumour in female dogs and, due to its similarities, is a valuable comparative model for human breast cancer. Kinase inhibitors have revolutionised the treatment of human breast cancer; their use in veterinary oncology remains marginal. **Aim:** This review summarises the current knowledge of kinase signalling pathways in CMC and assesses which kinase inhibitors approved for human use have potential in veterinary medicine. **Methods**: A systematic search of the PubMed database from 1985 to 2025 was performed, focusing on kinase-targeted therapies in both human and canine mammary carcinomas. Data were categorised according to molecular target, clinical approval status, and available preclinical or clinical veterinary evidence. **Results**: Key molecular pathways targeted by kinase inhibitors are conserved across species, supporting translational opportunities. In vitro studies demonstrate that palbociclib, alpelisib, everolimus, and lapatinib inhibit growth and signalling in CMC cell lines. Clinical trials have not been conducted. **Conclusions:** Approved kinase inhibitors for human use have untapped therapeutic potential in veterinary oncology. Translational research, including xenograft and organoid models, followed by clinical trials in dogs, is required. Gaining this knowledge could lead to targeted treatment for dogs while advancing comparative understanding of mammary cancer biology across species.

## Simple Summary

Canine mammary cancer is one of the most common cancers in females. In humans, drugs that block enzymes responsible for cell division, called kinases, have become an essential part of modern treatment, significantly improving survival. These drugs, approved for human use, can generally be adapted for veterinary use without the need to develop entirely new compounds. However, this article demonstrates that only a small number of these kinase inhibitors have been tested or used in dogs, despite evidence that the same biological pathways drive cancer growth in both species. To make the necessary progress, additional laboratory studies are needed to confirm the safety and activity of these drugs in canine cancer models, such as cell cultures and xenotransplantation, and to corroborate these findings in randomised clinical trials in dogs with spontaneous mammary cancer.

## 1. Introduction

Canine mammary cancer (CMC) represents one of the most frequently diagnosed neoplasms in female dogs. Depending on the studied population, mammary tumours account for up to 70% of all tumours in females, with malignancy rates ranging from 40% to over 80% [1,2]. This remarkable prevalence, combined with the spontaneous occurrence of these tumours and their molecular similarities to human breast cancer, makes the dog an important comparative model in translational oncology [3]. The histological diversity, hormonal dependency, and metastatic behaviour of CMC closely mirror those of human breast carcinoma [4,5].

The management of human breast cancer treatment has been revolutionised by the development of molecularly targeted therapies over the past decades. Notably, kinase inhibitors have reshaped therapeutic paradigms by modulating intracellular signalling pathways that drive cancer proliferation, survival, and metastasis [6]. Targeting the cyclin D/cyclin-dependent kinases 4 and 6 (CDK4/6)-retinoblastoma protein (RB) pathway (CDK4/6-RB), phosphatidylinositol 3-kinase (PI3K)/protein kinase B (Akt)/mammalian target of rapamycin (mTOR) signalling pathway (PI3K/AKT/mTOR), receptor tyrosine-protein kinase erbB-2 (HER2), and other kinases has significantly improved disease outcomes and survival in specific molecular subtypes of breast cancer [7,8,9,10]. In contrast, the therapeutic strategies of canine mammary tumours remain largely on conventional mastectomy, rarely supported with hormonal or adjuvant chemotherapy [11]. The limited progress in molecularly targeted therapies for CMC reflects several constraints. These include the limited availability of validated canine-specific drugs and the absence of clinical trials in the canine population. Nevertheless, a summary of genomic and proteomic data indicates that many of the signalling cascades dysregulated in human breast cancer are also aberrant in CMC, including the PI3K/AKT/mTOR axis [12] or the ERBB/HER family [13]. Such similarities provide compelling reasons to investigate the use of kinase inhibitors as potential therapeutic options in canine mammary tumours [14].

This review aims to map the current state of knowledge regarding kinase inhibitors relevant to the treatment of canine mammary cancer. It summarises existing data on kinase inhibitors currently used in the treatment of human breast cancer, reviews kinase signalling in canine mammary tumours, identifies drugs that have already been tested in the canine context, either in vitro or in vivo, and assesses the translational potential of incorporating kinase inhibition into veterinary oncology. By combining veterinary and human studies, the article highlights the potential of comparative oncology and demonstrates the feasibility and urgency of adapting human drugs for veterinary use.

## 2. Kinase Inhibitors: Historical Development, Chemical Classes, and Regulatory Milestones in Breast Cancer Therapy

The discovery and clinical application of kinase inhibitors (KIs) represent among the most significant achievements in modern oncology. This concept emerged in the late 1980s, when phosphorylation-dependent control of cell signalling was recognised as a key driver of cancer development. Aberrant activation of protein kinases, enzymes that transfer a phosphate group from ATP to serine, threonine, or tyrosine residues, has been associated with uncontrolled proliferation, resistance to apoptosis, and metastatic potential [6,15]. Early attempts to selectively modulate this activity were restricted by the high degree of conservation of the kinase’s ATP-binding pockets [16]. However, the 1990s saw rapid advances in structural biology and combinatorial chemistry, enabling the design of small-molecule inhibitors that target specific kinases with unprecedented precision [17]. Kinase inhibitors are a chemically diverse group but share standard pharmacophoric features, often mimicking ATP or allosterically modulating regulatory domains. They can be divided into type I inhibitors, which occupy the active ATP-binding site; type II inhibitors, which bind the inactive DFG-out conformation; and type III/IV allosteric inhibitors, which target regulatory pockets outside the catalytic cleft [18]. Most drugs approved for clinical use are small molecules (<600 Da), although there is increasing interest in drugs based on covalent and reversible inhibitors [19,20]. Some kinase inhibitors exhibit multikinase activity (e.g., toceranib, masitinib, dasatinib) [21], whereas others achieve remarkable selectivity for a single target (e.g., tucatinib for HER2) [22].

The timeline for the approval of kinase inhibitors in human breast cancer treatment regimens (Table 1) reflects parallel progress in understanding molecular and cellular mechanisms and in the development of knowledge for targeted drug design.

*Lapatinib* was the first kinase inhibitor approved for breast cancer, which shifted the cancer treatment paradigm in 2007. This small, orally administered molecule becomes an alternative to monoclonal antibody therapy in HER2-driven disease. It presented a multitargeted approach via dual inhibition of HER2 and EGFR [24]. Several years later (2012), the approval of *Everolimus* further advanced the concept of pathway blockade on metabolic signalling via the mechanistic target of rapamycin (mTOR), creating the first clinically confirmed example of targeting the PI3K/AKT/mTOR axis in hormone receptor-positive disease [25].

The endocrine therapy for HR^+^/HER2^−^ breast cancer was transformed between 2015 and 2017 by cyclin-dependent kinases 4 and 6 (CDK4/6) inhibitors (*Palbociclib*, *Ribociclib*, *Abemaciclib*) introduction. The direct influence of drugs on cell division improved progression-free and overall survival of affected individuals [26].

HER2-expressing breast cancers constitute a large group that is typically treated with the monoclonal antibody *Trastuzumab* [27]. However, due to the development of an immune response to this protein factor, second-generation HER2 inhibitors have also been developed: *Neratinib* and *Tucatinib*. In 2017, *Neratinib*, an irreversible pan-HER2 inhibitor, was approved for extended adjuvant therapy. Distinguished by its HER2 selectivity and penetration into the central nervous system, *Tucatinib* became the standard treatment for HER2^+^-positive brain metastatic breast cancer in 2020 [28,29].

Meanwhile, genomic studies of HR^+^/HER2-specific breast cancers identified *PIK3CA* gene mutations in approximately 40% of cases [30]. This explained the lack of response to treatment with the monoclonal antibody trastuzumab [31]. This discovery led to the approval of *Alpelisib* (2019), the first PI3Kα-specific inhibitor. This milestone was followed by *Capivasertib* (an AKT inhibitor, 2023) and *Inavolisib* (a new-generation PI3Kα inhibitor, 2024), both drugs aimed at addressing feedback activation and reducing the toxicity seen with earlier medications.

In addition to therapies specific to the molecular profile of breast cancer, the Food and Drug Administration (FDA) approved the combination of *Dabrafenib* and *Trametinib* for the treatment of BRAF-mutated breast cancer in 2022. The simultaneous effect on two distinct proteins in the MAPK/ERK signalling pathway is intended to inhibit the growth of cancer cells with a specific *BRAF* mutation. *Dabrafenib* inhibits the BRAF protein, whose overactivity is caused by mutations (V600E), while *Trametinib* is a selective inhibitor of the MEK1/2 protein. Although these molecular events are rare in breast tumours, the approval of this treatment regimen offers the opportunity to halt disease progression in vulnerable individuals [32,33].

In summary, the path from non-selective ATP-competitive inhibitors to precisely engineered molecules illustrates the evolution of kinase inhibitor pharmacology in breast oncology. Early drugs, such as *Lapatinib*, influenced HER2 and EGFR signalling pathways, and were primarily combined with capecitabine, a cytotoxic agent, to delay disease progression in trastuzumab-treated patients [34]. This generation confirmed the clinical relevance of dual kinase inhibition, but it was often limited by toxicity and the rapid emergence of resistance [35].

The approval of the second- and third-generation HER2 inhibitors *Neratinib* and *Tucatinib* has led to breakthroughs in selectivity and therapeutic integration. For example, *Tucatinib* demonstrated synergistic activity when combined with trastuzumab and capecitabine, yielding a durable intracranial response in HER2-positive metastatic breast cancer, including brain metastases (HER2CLIMB study) [36]. As an extended adjuvant therapy after trastuzumab, *Neratinib* leverages irreversible pan-HER inhibition to eradicate residual micrometastatic disease [37]. These treatment regimens represent a clear shift toward rational combination therapy, matching the pharmacodynamic profiles of kinase inhibitors with complementary mechanisms of antibody-mediated cytotoxicity or DNA-damage induction.

Similarly, in hormone receptor-positive, HER2-negative disease, progress has been achieved by simultaneously targeting endocrine signalling and cell cycle control. The introduction of the CDK4/6 inhibitors *Palbociclib*, *Ribociclib*, and *Abemaciclib* has redefined the standard of care by restoring sensitivity to oestrogen deficiency [38]. Each of these agents are currently FDA-approved in combination with aromatase inhibitors (letrozole or anastrozole) or fulvestrant, a selective oestrogen receptor degrader [39]. In clinical practice, these combination regimens not only extend progression-free survival by 8–12 months but also modulate resistance pathways by inhibiting cyclin D-dependent RB protein phosphorylation, thereby maintaining endocrine control [40,41,42,43].

The continued development of pharmaceuticals targeting the PI3K/AKT/mTOR axis has further expanded the possibilities for novel drug combinations. The PI3Kα-specific inhibitor Alpelisib, in combination with fulvestrant, became the first targeted therapy to counteract endocrine resistance caused by PIK3CA mutations (SOLAR-1 study) [44]. Similarly, the mTOR inhibitor *Everolimus*, in combination with the steroidal aromatase inhibitor exemestane, provided significant clinical benefit in patients who had previously failed nonsteroidal aromatase inhibitors (BOLERO-2 study) [45]. The recently approved ATP-competitive AKT1/2/3 inhibitor *Capivasertib* synergises with fulvestrant, providing durable control in tumours with AKT pathway abnormalities, while *Inavolisib* extends this principle by reducing the risk of hyperglycemia [46].

The combined therapeutic construct, a kinase inhibitor with a cytotoxic drug, an antibody, and a hormonal blockade, represents the culmination of decades of advances in knowledge and pharmacological refinements. With each generation, inhibitors not only have their selectivity and safety increased, but also their precision in complementing existing treatment methods. The current therapeutic regimen for human breast cancer thus represents a combination of chemical innovation and biomarker-based medicine, where synergies are meticulously predicted rather than discovered by chance. It is this therapeutic foundation, grounded in knowledge of the molecular mechanisms of carcinogenesis, which offers the most significant translational promise for comparative veterinary oncology. Applying these combined strategies to the treatment of naturally occurring mammary carcinoma in dogs could bridge the gap between experimental pharmacology and clinically relevant results across species.

## 3. Molecular Pathways Relevant to Kinase Inhibition in Canine Mammary Carcinoma

Tightly regulated kinase signalling cascades govern the proliferation and survival of mammary epithelial cells. In neoplastic transformation, these pathways become constitutively activated through mutations, amplifications, or loss of tumour suppressor control. Among the numerous kinases implicated in mammary carcinogenesis, three major networks, CDK4/6/RB, PI3K/AKT/mTOR, and HER2/EGFR (ErbB), play particularly critical roles and are already the mainstay of targeted therapies in human breast cancer.

### 3.1. The CDK4/6/RB Pathway

Dysregulation of the cell cycle machinery is a key hallmark of mammary carcinogenesis. In both human and canine cancers, the progression from G1 to S phase is critically regulated by cyclin-dependent kinase 4 and 6 (CDK4/6), which phosphorylate the retinoblastoma protein (RB1) [47,48]. This phosphorylation inactivates the inhibitory function of RB, allowing for E2F transcription factors to activate genes essential for DNA synthesis and entry into S phase [49,50]. Disruption of this checkpoint, for example, through overexpression of cyclin D1, amplification of CDK4, or loss of RB1, leads to uncontrolled cell proliferation and resistance to antiproliferative signals [51,52].In human breast cancer, particularly in luminal A and B subtypes, cyclin D1 amplification and RB1 signalling aberrations are frequently observed. These findings provide a biological rationale for developing selective CDK4/6 kinase inhibitors that restore control of this checkpoint by maintaining RB1 in a hypophosphorylated, active state. In canine mammary cancer, available molecular and histological evidence suggests that the CDK4/6/RB axis is functionally conserved. The CMCs are characterised by detectable cyclin D1 and nuclear RB protein expression, reflecting the luminal molecular subtypes in humans [53,54]. Cyclin D1 overexpression in CMCs correlates with higher histological grade and proliferative index, supporting its role as a driver of tumour aggressiveness [55].

In summary, the currently available evidence supports the hypothesis that CDK4/6 inhibition may be beneficial in canine mammary cancer, particularly in luminal subtypes with intact RB protein and elevated cyclin D1 levels. Incorporating these compounds into comparative oncology studies could provide new therapeutic options for dogs.

### 3.2. The PI3K/AKT/mTOR Pathway

Aberrant activation of the phosphatidylinositol 3-kinase (PI3K/AKT/mTOR) signalling pathway is among the most common oncogenic mutations in breast cancer. It contributes to uncontrolled growth, survival, and metabolic reprogramming of cancer cells. In human disease, this pathway integrates extracellular signals from receptor tyrosine kinases (RTKs) such as HER2 and IGF1R, converting them into phosphorylation signals that regulate cell proliferation and apoptosis. Mutations in the *PIK3CA* gene (encoding the p110α catalytic subunit), loss of PTEN, and activation of AKT are recurrent mechanisms that drive constitutive pathway stimulation [56,57].

In canine mammary carcinoma, similar molecular changes have been reported. Studies of PIK3CA protein/gene function at the nucleotide sequence level have revealed frequent hotspot mutations (H1047R, E545K, E542K) and loss of PTEN expression, which, in immunohistochemical studies, have been associated with elevated phospho-AKT levels and subsequent mTOR activation [58,59,60,61]. PIK3CA mutations have been confirmed in approximately 25–30% of CMC cases [62], mirroring the pattern of mutation frequency in human breast cancers. These findings provide a compelling rationale for research on PI3K/AKT/mTOR inhibitors in veterinary oncology.

Taken together, these observations position the PI3K/AKT/mTOR cascade as a promising cross-species therapeutic axis. Its well-documented role in tumour progression, availability of targeted inhibitors, and the presence of analogous genetic alterations in CMC make it one of the most attractive molecular pathways for comparative oncologic drug development.

### 3.3. HER2/EGFR Pathway and the ERBB Family

The HER/ErbB family comprises human epidermal growth factor receptors that exhibit tyrosine kinase activity. It contains four receptors: EGFR (HER1), HER2 (ErbB2), HER3, and HER4, which play crucial roles in regulating cell proliferation, survival, and differentiation. Dysregulation of this receptor family, usually related to HER2/ErbB2 overexpression by gene amplification, defines one of the most aggressive human breast cancer subtypes, affecting approximately 15–20% of cases [63]. HER2 lacks a known ligand and exerts its oncogenic function primarily through homodimerization or constitutive dimerisation with other ErbB receptors, activating downstream PI3K/AKT/mTOR and RAS/MAPK signalling cascades [64,65].

Dysregulation of HER2 expression has also been observed in canine mammary carcinoma (CMC). Unfortunately, the prevalence of these mutations and their prognostic significance remain unclear, primarily due to observed interstudy variability and differences in antibody specificity [66,67]. Initial immunohistochemical analyses demonstrated HER2 expression in 17–40% of CMC, often correlating with high histological grade and poor prognosis [1,68,69,70]. Subsequent molecular studies revealed gene amplification in some of the studied cases, strengthening the biological analogy with HER2-positive human breast cancer [68,71]. Reference [68] proposed diagnostic thresholds analogous to the human ASCO/CAP criteria, finding that true HER2 overexpression (3+) occurs in approximately 10–15% of canine mammary carcinomas. They also found that HER2 overexpression in dogs is associated with gene amplification but is not associated with cancer behaviour. Recent genomic studies further indicate that HER2 dysregulation in dogs frequently co-occurs with alterations in PI3K pathway genes, suggesting parallel oncogenic networks across species [62].

## 4. Kinase Inhibitors in Veterinary Oncology: Current Use and Evidence in Canine Mammary Carcinoma (CMC)

Over the past two decades, kinase inhibitors have fundamentally changed the landscape of human oncology, providing highly selective drugs that target disrupted signalling pathways. However, their adaptation in veterinary oncology remains marginal. Despite the widespread success of small-molecule inhibitors in human cancer therapy, only three drugs, toceranib phosphate (Palladia^®^), masitinib (Kinavet^®^/Masivet^®^), and imatinib (Gleevec^®^) have reached the veterinary market. Toceranib, developed by Pfizer, was the first targeted anticancer drug approved by the FDA for dogs in 2009. It acts as a multi-targeted tyrosine kinase inhibitor, blocking KIT, VEGFR2, and PDGFRβ [72], and is indicated for the treatment of recurrent, unresectable cutaneous mast cell tumours with or without regional lymph node metastasis. Clinical trials have tested the drug in spontaneous tumours, including mixed mammary cancers and soft-tissue sarcomas [73]. Masitinib, developed by AB Science and approved by the European Medicines Agency in 2010, shares a similar inhibitory profile against KIT, PDGFR, and Lyn kinases, and is used for nonresectable grade II and III mast cell tumours [74], extending median overall survival to 617 days in the masitinib-treated group compared to 322 days in the placebo group [75]. A third agent, imatinib, although never formally approved for veterinary use, has been extensively studied in dogs with KIT-mutated mast cell tumours and gastrointestinal stromal tumours, demonstrating positive clinical effects [76,77,78], such as imatinib adjuvant therapy for an incompletely resected multilobular tumour of bone in a dog, showing good functional outcome for 306 days [79].

Despite these milestones, no kinase inhibitor has yet been approved for the treatment of epithelial cancers, including canine mammary carcinoma (Table 2). However, the last decade has seen an increase in preclinical research on kinase signalling in CMC, demonstrating similarities in the activation of many oncogenic pathways targeted in human breast cancer. Three signalling axes: CDK4/6-RB, PI3K/AKT/mTOR, and HER2/EGFR, have been extensively studied, and knowledge about their dysfunction can be successfully applied to identify potential therapeutic targets.

### 4.1. The CDK4/6/RB Pathway

To date, the only drug being investigated for use in veterinary oncology is the selective CDK4/6 inhibitor *Palbociclib*, approved by the FDA in 2015 for the treatment of advanced hormone receptor-positive and HER2-negative human breast cancer. The in vitro study demonstrated that *Palbociclib* induces a dose-dependent reduction in proliferation, inhibits E2F target gene expression, and reduces clonogenicity in canine mammary cancer cell lines (P114 and CF41), closely mirroring its activity in human luminal breast cancer [80]. These findings confirmed that the RB-dependent checkpoint is functionally conserved in CMC. Furthermore, a clinical trial, “Pilot Study Evaluating Palbociclib in Dogs with Melanoma, Squamous Cell Carcinoma, Mammary Carcinoma, or Anal Sac Adenocarcinoma,” was initiated in 2024, representing the first veterinary clinical trial for this drug [84]. A study also evaluated four canine melanoma cell lines and found a potential positive effect of this drug in three [47]. Preclinical studies during the drug’s registration process for human use evaluated 0.2 mg/kg/day of *Palbociclib* as a safe dose in healthy dogs [85].

However, despite encouraging data on dysregulation of this pathway in canine mammary cancer, no preclinical studies on CMC tumour lines, nor clinical studies evaluating other CDK4/6 inhibitors (*Ribociclib*, *Abemaciclib*) for the possible treatment of dogs with spontaneous mammary carcinoma, have yet been published.

### 4.2. The PI3K/AKT/mTOR Signalling Axis

The PI3K/AKT/mTOR pathway, one of the most frequently altered signalling cascades in human breast cancer, is also dysregulated in CMC. Mutations in the *PIK3CA* gene, particularly in the H1047R and E545K hotspots, and loss of PTEN expression have been documented in up to one-third of canine mammary tumours [86]. The PI3Kα-specific inhibitor Alpelisib suppresses AKT activation and triggers apoptosis in *PIK3CA*-mutant canine primary mammary cancer cell lines [81]. The study of canine hemangiosarcoma cell lines (HAS) suggests that Alpelisib may be highly effective against *PIK3CA* mutations, which occur frequently in canine HAS [87]. The mTOR signalling pathway may be a potential therapeutic target. In a study on the CTBp adherent and sphere-forming canine mammary tumour cell line, phosphorylated mTOR decreased by *Everolimus* exposure. In mice transplanted with CTBp-derived spheres, *Everolimus* treatment significantly reduced tumour volume compared to control [82]. Moreover, *Everolimus* combined with carboplatin chemotherapy may have activity in canine melanoma [88].

These findings provide a direct translational link between human and canine mammary carcinogenesis. These data pertain to only two of the four kinase inhibitors approved for the treatment of human breast cancer. No information is available on *Capivasertib* and *Inavolisib*. However, as with CDK4/6 inhibitors, no prospective in vivo studies have assessed the clinical activity of *Alpelisib* and *Everolimus* as PI3K or mTOR inhibitors in dogs with spontaneous CMC.

### 4.3. HER2/EGFR and the ERBB Family

Aggressive molecular subtypes of breast cancer in humans coexists with *HER2* amplification and overexpression. In dogs, *HER2* overexpression has been observed in 15–20% of CMCs, though reported frequencies vary widely due to differences in antibody validation and scoring protocols [63]. Established in 1998, trastuzumab was the first antibody-based targeted treatment for a solid tumour, dramatically improving survival in HER2-positive disease [89]. Despite the proven homology of the *HER2* gene between humans and dogs [90], and its demonstrated inhibitory effect on cell growth in vitro and in vivo in nude mice [91], this monoclonal antibody has not been adopted for veterinary use. The most crucial argument against its introduction was the potential for dogs to mount an immune response to the humanised antibody, leading to severe allergic or hypersensitivity reactions [92]. For this reason, veterinary cancer treatment should prioritise kinase inhibitors targeting this cellular pathway. Three drugs are FDA-approved for the treatment of human breast cancer: *Lapatinib*, *Tucatinib*, and *Neratinib*. Only the earliest approved for human use, *Lapatinib*, was evaluated for canine mammary cancer. In vitro, as a dual HER2/EGFR inhibitor, it exhibited a dose-dependent reduction in cell viability, decreased phosphorylation of HER2 and AKT, and induced apoptosis in primary CMC cell lines [83]. These effects mirrored those seen in HER2-positive human breast cancer cell lines, confirming mechanistic conservation. The *Lapatinib* exerts anti-tumour effects on canine transitional cell carcinoma (TCC) cell lines [93], as well as the muPC canine prostatic carcinoma cell line [94] in a nude mouse xenographic model. In clinical research, *Lapatinib* showed encouraging durable response rates, survival, and tolerability, supporting its therapeutic use for untreated advanced urothelial carcinoma in dogs [95]. It was estimated that a dose of 35 mg/kg/day, administered for no more than 8 weeks, was safe for healthy dogs [96].

Another kinase inhibitor, *Neratinib*, has not been evaluated in canine mammary cancer in vivo or in vitro. However, a Phase II study of *Neratinib* in dogs with Pulmonary Adenocarcinoma was conducted [97], and in vitro studies on recurrent *HER2 (ERBB2)* mutations occurring in canine pulmonary adenocarcinoma (cPAC) exhibited significantly higher sensitivity to the HER2 inhibitors *Lapatinib* and *Neratinib* [98]. There are no scientific reports on the use of *Tucatinib* in the treatment of dog mammary cancer, or indeed any cancer.

Although in vivo studies or clinical trials in dogs remain insufficient, the cumulative evidence supports the concept that HER2/EGFR signalling is a viable therapeutic target in canine mammary carcinoma.

### 4.4. BRAF Mutation

The serine/threonine kinase v-Raf murine sarcoma viral oncogene homologue B1 (BRAF) plays a crucial role in the MAPK signalling cascade, which regulates cell proliferation, differentiation, and survival. Constitutive activation of the BRAF pathway, driven by *BRAF* mutations, leads to uncontrolled cellular growth. The *BRAF* mutation occurs in approximately 8% of all human cancers, with high prevalence in malignant melanoma (~50%), papillary thyroid carcinoma (~40%), and colorectal carcinoma (~10%) [99,100]. Although breast cancer is uncommon (approx. 1%) [101], the FDA granted tumour-agnostic approval for *Dabrafenib* and *Trametinib* combination in solid tumours with BRAF V600E mutation [32]. The BRAF V595E mutation, which results in constitutive ERK1/2 phosphorylation and downstream proliferative signalling, independent of upstream RAS activation, occurs in approximately 65–85% of canine urothelial carcinomas (UCs) and 30–40% of prostatic carcinomas [102,103]. Significantly, noninvasive diagnostic assays for urine-derived DNA were developed to detect the BRAF^V595E mutation, with >95% specificity for canine urothelial and prostatic carcinomas [103]. The low frequency of the BRAF^V595E mutation in canine mammary cancer has been confirmed [104], which is consistent with data for human breast cancer.

Of the *Dabrafenib* and *Trametinib* combinations, only *Trametinib* has been evaluated to a limited extent for use in dogs. In phase II clinical studies, the dose of 0.5 mg/m^2^/day was considered safe and recommended for dogs with cancer [105]. Studies have shown *Trametinib’s* effectiveness in the growth inhibition of canine oral squamous cell carcinoma [106]

## 5. Adverse Events Associated with Kinase Inhibitors: Translational Insights from Human Oncology and Preclinical Canine Data

The safety profile of kinase inhibitors is a key factor in their translational potential in veterinary oncology. In human medicine, adverse events (AEs) associated with these drugs are well characterised in extensive Phase I–III clinical trials, encompassing both acute and chronic toxicities in various therapeutic settings. However, available safety data for kinase inhibitors in dogs come almost exclusively from preclinical toxicology studies, where the species serves as a laboratory model rather than a clinical patient.

Therefore, most published information concerns single-dose (acute) toxicity studies, repeated-dose toxicity studies (2–4 weeks or 13 weeks), or chronic exposure studies (6–9 months), in which doses or study durations are often several times higher than those intended for therapeutic use in humans. These studies, primarily designed for regulatory safety assessment in human drug development, cannot be directly extrapolated to veterinary oncology without careful consideration of dose, formulation, and clinical context. Nevertheless, they provide valuable preliminary insights into organ sensitivity, pharmacokinetic behaviour, and toxicity patterns associated with drug classes, particularly gastrointestinal disorders, hepatotoxicity, myelosuppression, and dermatological reactions.

### 5.1. The CDK4/6/RB Pathway

*Palbociclib* is administered to humans at a standard dose of 1.8 mg/kg/day (125 mg/day) in 28-day cycles (21-day on and 7-day off regimen), reflecting the need for cyclical recovery from predictable hematologic suppression. The PALOMA-1–3 trials revealed hematologic toxicities as the dominant AE. Occurring in up to ~80–90% of patients (any grade), with grade 3–4 reported in approximately 55–65%, neutropenia was the most frequent and dose-limiting toxicity. Other adverse events frequently observed across studies included leukopenia, anaemia, nausea, fatigue, stomatitis, alopecia, and mild infections. Most AEs are managed effectively by reducing the dose to 1.8 mg/kg/day (100 mg/day) or 1 mg/kg/day (75 mg/day), without compromising therapeutic efficacy or progression-free survival. Overall, the human safety profile of palbociclib is characterised by reversible myelosuppression, non-hematologic toxicities, and a low incidence of severe complications, which can be managed by dose reduction (1.42 mg/kg/day (100 mg/day), 1 mg/kg/day (75 mg/day) [40,107,108]. 

In toxicology studies in beagle dogs, *Palbociclib* was administered at doses ranging from 0.2 to 3 mg/kg/day over repeated cycles (3 weeks on, 1 week off), with a 39-week long-term exposure. At lower to intermediate doses (≤2–3 mg/kg/day), dogs exhibited dose-dependent, reversible suppression of bone marrow and lymphoid tissues, as well as mild gastrointestinal and reproductive (testicular) toxic effects. Neutropenia was present even at clinically relevant exposures (0.6–2 mg/kg/day), with profound changes occurring at 3 mg/kg/day. These exposures are comparable to standard human dosing (1.8 mg/kg/day), suggesting that therapeutic doses for dogs could theoretically align with human exposure. In contrast, acute and subacute toxicity studies (5–30 mg/kg/day) revealed serious cardiopulmonary and respiratory effects. Therefore, if *Palbociclib* was to be repurposed for veterinary use, a conservative dosing strategy, likely ≤1–2 mg/kg/day with an intermittent schedule and close hematologic monitoring, should be required [85,107].

In humans, *Ribociclib* is administered in a 28-day cycle (21 days on and 7 days off) at a recommended dose of 8.6 mg/kg/day (600 mg/day). The pivotal MONALEESA trials consistently identified myelosuppression as the primary dose-limiting toxicity. Any grade neutropenia occurred in 74% of patients, with grade 3–4 in 59%. Leukopenia, lymphopenia, anaemia and mild infections were also frequently observed. Non-hematologic toxicities included hepatobiliary injury (ALT/AST elevations, grade 3–4 ALT in ~9%), QTc prolongation (~4–5%), fatigue, nausea and mild gastrointestinal symptoms. Notably, toxicities are reversible and manageable by reducing the dose to 5.71 mg/kg/day (400 mg/day) or 2.85 mg/kg/day (200 mg/day) [43,109]. In dogs, all available data stem from toxicology studies conducted for regulatory approval. In single-dose oral studies, beagle dogs tolerated doses up to 100 mg/kg, although salivation, vomiting, reduced activity, and erythema appeared at ≥25–50 mg/kg. Intravenous injection identified the approximate maximum tolerated dose (5 mg/kg). In repeat-dose studies (1, 3, 10, 20 mg/kg/day), a schedule that mirrors human treatment (3 weeks on/1 week off), dogs exhibited a toxicity profile broadly similar to humans but emerging at substantially higher mg/kg levels. The primary target organs were bone marrow, lymphoid tissues, liver (proliferative and cholestatic changes), and gonads (atrophy). Decreased intestinal mucosal thickness, reduced bone formation, and QT interval prolongation were also reported in short-term studies [109]. Overall, *Ribociclib* demonstrates a highly concordant cross-species toxicologic signature, characterised by reversible cytostatic suppression of the bone marrow and lymphoid compartments. However, dogs were exposed to mg/kg doses several-fold higher than human therapeutic levels, as is typical for toxicology programmes. These findings suggest that if *Ribociclib* were to be explored in canine oncology, initial dosing would need to remain well below toxicology-tested levels and closer to human mg/kg equivalents, coupled with regular hematologic monitoring and ECG assessment.

In humans, *Abemaciclib* is approved at two main doses, in combination with endocrine therapy (e.g., fulvestrant or a non-steroidal aromatase inhibitor). The recommended dose is 4.3 mg/kg/day (300 mg/day). In contrast, in monotherapy, the dose is 5.7 mg/kg/day (400 mg/day) and is administered continuously without interruption. The MONARCH 1–3 clinical trials found that the most frequent AE is diarrhoea (82–90%, grade 3 in ~10–20%), irrespective of whether it is used alone or in combination, and that it is typically manageable with antidiarrheal prophylaxis, dose reduction, or interruption. The hematologic toxicity is expressed in neutropenia, as any grade is seen in the majority of treated individuals. Other common adverse events include anaemia, fatigue, nausea, and decreased appetite. Overall, the clinical safety profile is characterised by predictable and reversible gastrointestinal and hematologic toxicity, and can be controlled by dose modification (2.8 mg/kg/day (200 mg/day) and 1.8 mg/kg/day (100 mg/day)) [41,110,111,112]. In dogs, available information comes from toxicology studies in beagle dogs. In a 1-month study, animals received 10 mg/kg/day orally, and key findings included liquid faeces, vomiting, weight loss, severe small- and large-intestinal lesions (villous/mucosal atrophy, crypt necrosis, haemorrhage), and bone marrow hypocellularity. In the 3-month dog study, doses up to 3 mg/kg/day did not produce overt intestinal lesions, although at ≥0.3 mg/kg/day, animals exhibited decreased body weight. In dose-repeat studies, dogs produced dose-dependent intestinal toxicity, bone-marrow suppression and lymphoid depletion, along with testicular toxicity at ≥0.3–3 mg/kg/day. Hematologic changes mirrored the human pattern and included reductions in lymphocytes, neutrophils, monocytes, platelets and red blood cells. Toxicokinetic data from the 3-month studies indicated that the maximum tolerated exposure (AUC) was approximately half of the human maximal exposure, at 5.7 mg/kg/day. In contrast, the lowest observed adverse effect level (LOAEL) was observed at exposures near or below the human therapeutic range, particularly for bone marrow and testes [110].

Taken together, these data show that *Abemaciclib* exhibits a highly consistent cross-species toxicity profile, dominated by gastrointestinal injury and cytostatic myelosuppression, with clinically relevant toxicity occurring in dogs at mg/kg doses in the same order of magnitude as, or even below, human therapeutic doses. For translational application, any investigational dosing would need to remain at or below human mg/kg equivalent levels (probably ≤1–2 mg/kg/day to start), with strict monitoring of diarrhoea, body weight, hematologic parameters and, in males, fertility-related endpoints.

### 5.2. The PI3K/AKT/mTOR Signalling Axis

In humans, *Alpelisib* is approved at a dose of 4.3 mg/kg/day (300 mg/day) for use in combination with fulvestrant until disease progression or toxicity. In the phase III SOLAR-1 trial, the toxicity profile was dominated by on-target metabolic effects and gastrointestinal or cutaneous toxicity. The most frequent adverse events were hyperglycemia (63.7%, grade 3–4 in 36.6%), diarrhoea (57.7%, grade 3–4 in 6.7%), nausea (44.7%), decreased appetite (35.6%), and rash (35.6%, grade 3–4 in 9.9%). Overall, the human safety profile is characterised by predictable, mechanism-based metabolic toxicity (hyperglycemia, dyslipidemia), together with rash and diarrhoea, which are largely manageable through prophylactic or reactive use of antidiabetic and antidiarrheal drugs, dose reductions (3.6 mg/kg/day (250 mg/day) and 2.8 mg/kg/day (200 mg/day) and interruption [44,113].

*Alpelisib* has been studied in repeat-dose GLP toxicology studies in beagle dogs conducted for human drug development. In a 4-week study with doses 2, 5, and 15 mg/kg/day, significant findings included body-weight loss starting at 2 mg/kg/day, dose dependent disturbances in glucose and insulin homeostasis at ≥2 mg/kg/day, and atrophic changes in the epithelium of the oral mucosa, tongue, oesophagus, larynx, and skin, as well as in the gastrointestinal mucosa and lymphoid tissues at 15 mg/kg/day. One 15 mg/kg/day dose, the test was ended because of severe weight loss, or acute intestinal intussusception. Insulin levels increased by 41–62% at 5 mg/kg/day and 62–139% at 15 mg/kg/day, accompanied by variable hyperglycemia and elevated triglycerides and cholesterol, confirming a strong on-target metabolic effect of PI3Kα inhibition in dogs. Decreases in pituitary, spleen and thymus weights (~30–35%) and dose-dependent reductions in ovary, uterus and prostate weights (up to ~40–50%) were also documented, along with uterine atrophy at the highest dose. In a 13-week study using 0.2, 1.0, and 5.0 mg/kg/day, *Alpelisib* was described as “generally well tolerated” in terms of overt clinical signs, ECG, and ophthalmologic findings. Nevertheless, even at these lower doses, disruption of glucose and insulin homeostasis, body weight gain (especially at 5 mg/kg/day), and minor postmortem changes in the tongue and lymphoid tissues of high-dose animals were observed. All findings were fully or partially reversible after a 4-week treatment-free period in both tests [113,114].

Taken together, these data show that *Alpelisib* produces broadly similar, mechanism-driven metabolic and mucocutaneous toxicities in humans and dogs. Nevertheless, in dogs, metabolic perturbations (hyperinsulinemia, dysglycemia, weight loss) and mucosal/skin changes appear at doses (0.2–5 mg/kg/day) that are at or below the human dose (4.3 mg/kg/day). From a translational perspective, this suggests that if *Alpelisib* were to be explored in canine oncology, any investigational dosing would need to start well below the human mg/kg-equivalent, with rigorous monitoring of body weight, fasting glucose, insulin, and lipid profiles, and close attention to oral, cutaneous and gastrointestinal integrity.

*Inavolisib* is administered at a low dose of 0.12 mg/kg/day (9 mg/day) in combination with *Palbociclib* and fulvestrant. The AEs for *Inavolisib* are dominated by hyperglycaemia (~59%), stomatitis (~51%), diarrhoea (~48%), and rash (~25%), together with laboratory abnormalities such as increased glucose and creatinine and decreased electrolytes, as evaluated in the INAVO120 trial. These events were generally manageable with dose reduction (up to 0.04 mg/kg/day (3 mg/day)) [115,116].

In dogs, *Inavolisib* has not been evaluated clinically, and all available information derives from regulatory repeat-dose toxicology studies in beagles. These studies identify the eye lens as a uniquely sensitive target organ. Lens fibre swelling occurred at ≥0.3 mg/kg/day, and cortical vacuolation at ≥1 mg/kg/day. Notably, the ocular changes observed in dogs were reversible following a 12-week recovery phase. Other toxicologic findings in dogs were consistent with PI3Kα pathway inhibition, disturbances in glucose-insulin regulation, mild metabolic changes, and lymphoid atrophy, but were observed primarily at higher exposures [116].

Taken together, these data indicate that *Inavolisib* exhibits a narrower safety margin in dogs than many other kinase inhibitors, owing to ocular sensitivity at exposures near the human therapeutic range. If the drug were ever considered for translational evaluation in canine mammary carcinoma, any investigational dosing would need to remain well below human mg/kg equivalents, with mandatory ophthalmologic surveillance and careful monitoring of metabolic parameters.

*Capivasertib*, in combination with fulvestrant, is administered at 11.4 mg/kg/day (800 mg/day) in a 4-week cycle (4 days on/3 days off). In the CAPItello-291 trial, the safety profile was dominated by on-target metabolic and gastrointestinal toxicities, with hyperglycaemia, diarrhoea and rash, superimposed on the background toxicity of fulvestrant. Hyperglycaemia of any grade was reported in about 14–15% of patients (grade 3–4 in 2–3%). Diarrhoea occurred in 67.3% (grade 34 in 9.3%). Cutaneous adverse reactions, including maculopapular rash and erythema multiforme, were reported in 47% (grade 3–4 rash in 17%) of patients. Hypertension, nausea, fatigue and decreased appetite also occur. Overall, clinical toxicities are dose-dependent but generally manageable with dose reductions (to 9.1 mg/kg/day (640 mg/day) and 5.71 mg/kg/day (640 mg/day)), intermittent schedule, and supportive care [46,117,118,119]. Safety pharmacology and GLP repeat-dose toxicology studies in beagles were conducted to support drug development. In single oral doses of 5, 30 and 40 mg/kg in cardiovascular safety studies, produced haemodynamic effects in dogs, decreased heart rate, transient reductions in systolic and diastolic blood pressure, and sustained prolongation of QTc, together with increased indices of left-ventricular contractility and rises in blood glucose and insulin concentrations. In a 1-month repeat-dose study (3, 10, 30 mg/kg/day), echocardiography demonstrated decreases in systolic left-ventricular diameter, increases in ejection and shortening fractions, and reductions in cardiac output at doses ≥10 mg/kg and QTc prolongation at 30 mg/kg. Longer-term dog studies (up to 9 months) identified a broader spectrum of target tissues. At doses ≥10 mg/kg/day, *Capivasertib* produced dose-dependent reduced food intake, weight loss, dysregulation of glucose-insulin homeostasis, and histopathological changes in the pancreas, liver, thymus and adrenal gland. Particularly notable were degenerative lesions in the male reproductive tract (testes, epididymides and prostate), which appeared at ≥15 mg/kg/day and did not fully resolve after a 4-week recovery period, indicating a potential risk of persistent infertility at higher exposures [117,118].

Taken together, the preclinical and clinical data indicate that *Capivasertib* produces a largely consistent pattern of toxicity across both species. In humans, this manifests as hyperglycemia, diarrhoea, and rash; in dogs, it manifests as metabolic disturbances, cardiovascular effects (including QTc prolongation and contractility abnormalities), and irreversible damage to male reproductive organs, occurring slightly above the therapeutic dose in humans. However, it is essential to emphasise that the drug was administered continuously, not 4 days on and 3 days off, as in humans. For future translational applications in canine oncology, these results suggest that doses lower than the simple equivalent of the human mg/kg dose will be required, with close monitoring of cardiovascular function, glucose and insulin levels, and male reproductive health, as is performed in human oncology.

*Everolimus* is recommended at a dose of 0.14 mg/kg/day (10 mg/day), taken continuously until disease progression or unacceptable toxicity. In the pivotal BOLERO-2 trial, the toxicity profile was dominated by stomatitis (~67%), infections (~50%), rash (~39%), fatigue (~36%), diarrhoea (~33%) and decreased appetite (~30%), with grade 3–4 adverse reactions occurring only in ≥2% of patients. The most frequent grade 3–4 laboratory abnormalities were lymphopenia, hyperglycemia, anaemia, hypokalemia, and elevations in AST/ALT and triglycerides. Non-infectious pneumonitis was reported in up to 19% (grade 3–4 in ~4%) of patients. The AEs are manageable through dose reductions to 0.07 mg/kg/day (5 mg/day), dose interruptions and supportive measures such as topical corticosteroid mouthwashes and rigorous infection monitoring [45,120,121]. In contrast, available data on dogs indicate that Everolimus has low acute toxicity in beagles. In a dose-escalation study in one male and one female beagle, oral doses of 2, 6 and 18 mg/kg administered on three consecutive days did not identify a maximum tolerated dose and produced no dose-limiting clinical signs, consistent with a substantial acute safety margin relative to the human oncologic dose (0.14 mg/kg/day) [122]. Long-term and repeat-dose toxicology studies were conducted in rats and non-human primates. In dogs, more relevant toxicity data come from hematopoietic stem-cell transplantation models, in which everolimus was administered as part of pre- and post-transplant immunosuppression in combination with cyclosporine or mycophenolate mofetil. In these studies, dogs experienced substantial regimen-related toxicity at a dose of 0.25 mg/kg/day, including gastrointestinal signs (diarrhoea, weight loss, anorexia), cytopenias and infectious complications consistent with profound immunosuppression. Importantly, these transplant protocols were designed to achieve a potent immunosuppressive effect by *Everolimus* combined with other myelosuppressive or nephrotoxic agents, rather than to define a chronic “oncologic” therapeutic window [123].

Taken together, human and dog data indicate that *Everolimus* exhibits a broadly consistent toxicity profile across species, characterised by immunosuppression, mucosal damage, metabolic disruption, and reproductive toxicity, but with relatively sparse data on repeated-dose studies in dogs compared with rodents and primates. These observations suggest that dogs are unlikely to be more sensitive than humans; however, the lack of systematic dose-escalation oncology studies in dogs means that the accurate therapeutic index in dogs for chronic anticancer drug use remains undefined, and any attempt to use *Everolimus* for the treatment of mammary carcinoma in dogs would require careful, prospectively monitored phase I studies, with particular attention to mucosal, infectious, metabolic, and reproductive endpoints.

### 5.3. HER2/EGFR and the ERBB Family

*Lapatinib* is recommended for humans at an 18 mg/kg/day (1.250 mg/day) dose in combination with capecitabine from day 1 to 14 in 3-week cycles, or at a 21 mg/kg/day (1.500 mg/day) dose in combination with letrozole for postmenopausal women with HR^+^/HER2^+^ metastatic disease. The most frequent any grade AE in lapatinib and capecitabine combination were diarrhoea (45–60%, grade 3–4 in 8–13%), palmar-plantar erythrodysesthesia (hand-foot syndrome, 50–60%, grade 3–4 in 9–14%), rash (33%, grade 3 in 1–5%), nausea, vomiting, fatigue, hepatotoxicity (elevations in ALT, AST, alkaline phosphatase, bilirubin, rare liver injury events) is a crucial labelled risk [124,125,126]. Overall, the human safety profile is defined by gastrointestinal and dermatologic toxicity plus a recognisable hepatobiliary signal, largely manageable with dose reduction (10.7 mg/kg/day (750 mg/day) with capecitabine and 14.3 mg/kg/day (1000 mg/day) with letrozole) interruption, antidiarrhoeal therapy and regular liver-function monitoring [35,127]. In dogs, *Lapatinib* has been evaluated in both regulatory toxicology studies in healthy beagles and early translational oncology trials, providing a relatively rich comparative dataset. The single oral toxicity test at doses of 50, 150 and 500 mg/kg/day in beagle dogs produced no significant changes in behaviour, neurology or autonomic function over seven days. Although blood-pressure elevations were observed at 150–500 mg/kg/day and at a 50 mg/kg/day dose, an isolated ventricular extrasystole occurred in one animal, indicating cardiovascular sensitivity at very high exposures. In 13-week repeat-dose studies with 10, 40 and 160 mg/kg/day doses, in the 160 mg/kg group, animals exhibited decreased activity, dehydration, salivation, loose faeces, oral and paw ulcerations, vomiting, weight loss, reduced food intake, and increased white blood cells (neutrophils, monocytes), bilirubin, bile acids, alkaline phosphatase and ALT elevation. In a 39-week study using 10, 40 and 100 mg/kg/day, two high-dose dogs were euthanised in the final weeks of the study, target organs were affected (gastrointestine, liver, skin, lymphoid tissues, adrenals), and substantial reversibility during recovery occurred [96,128].

Beyond toxicology data from clinical trials, a dedicated tolerability study in six healthy beagles established a maximum tolerated dose of 35 mg/kg/day orally for 8 weeks. In the study, one dog developed dose-limiting grade 3 toxicity (>15% body-weight loss) at a 40 mg/kg/day dose, and at 35 mg/kg/day, three of six dogs developed grade 3 elevations in alkaline phosphatase, confirming a clinically relevant hepatobiliary liability at doses roughly 2-fold the human mg/kg exposure [96]. A prospective, non-randomised clinical trial in dogs with naturally occurring urothelial carcinoma used lapatinib at 20–30 mg/kg/day orally in combination with piroxicam 0.3 mg/kg/day. The regimen was associated with improved response rates and survival compared with piroxicam alone, and adverse events were mostly grade 1–2, with dose reductions required in ~9% of dogs. Elevations in ALT and ALP were significantly more frequent in the lapatinib group, and dermatologic changes (hyperpigmentation, pruritus, ulceration, alopecia) occurred in about 11% of treated dogs, typically without necessitating discontinuation [95,128].

Taken together, these data indicate that *Lapatinib* induces a similar set of toxicities in humans and dogs, including diarrhoea, skin reactions, and hepatobiliary toxicity. However, the absolute mg/kg doses tolerated by dogs in toxicology studies and early clinical trials (20–35 mg/kg/day) are higher than the dose per kilogram of body weight in humans (18–21 mg/kg/day). For translational use in veterinary oncology, existing data in urothelial carcinoma suggest that daily doses in the range of 20–30 mg/kg are feasible with close biochemical and clinical monitoring. However, hepatobiliary function, gastrointestinal tolerance, and dermal toxicity would require systematic monitoring, reflecting the safety concerns identified in human breast cancer therapy.

*Tucatinib* is approved at a dose of 8.6 mg/kg/day (600 mg/day) in combination with trastuzumab and capecitabine on 3-week cycles for human use. In the HER2CLIMB trial (tucatinib, trastuzumab, and capecitabine), a toxicity profile dominated by diarrhoea, palmar-plantar erythrodysaesthesia, hepatotoxicity and gastrointestinal adverse events was seen [129]. The most common AEs (≥20%) in the *Tucatinib* treatment were diarrhoea (81%, 3–4 in 12.5%), palmar-plantar erythrodysaesthesia (63%, grade 3 in 13%), nausea (58%), hepatotoxicity (42%, grade 3–4 in 9%), vomiting (36%), stomatitis (32%), decreased appetite (25%), anaemia (21%) and rash (20%). Laboratory abnormalities were consistent with increased bilirubin (47%, grade 3–4 in 1.5%), ALT (46%, grade 3–4 in 8%) and AST (43%, grade 3–4 in 6%). Overall, *Tucatinib’s* clinical safety profile is defined by dose-dependent diarrhoea and hepatobiliary toxicity, largely manageable with dose reductions (7.1 mg/kg/day (500 mg/day), 5.7 mg/kg/day (400 mg/day) and 4.28 mg/kg/day (300 mg/day)), antidiarrhoeal therapy and close monitoring of liver function [36,130,131].

In contrast to other HER2-targeted agents, no dedicated dog toxicology programme is reported for *Tucatinib* in the available regulatory documentation. The non-clinical safety data have been characterised primarily in rats and cynomolgus monkeys with very high mg/kg exposures (≥90–200 mg/kg/day) [130].

Collectively, data indicate that *Tucatinib*, at clinically relevant human doses (8.6 mg/kg/day (600 mg/day)), is associated with gastrointestinal and hepatobiliary toxicity superimposed on the background AE profile of trastuzumab and capecitabine. Because dogs were not used as a regulatory toxicology species for *Tucatinib*, there is no direct canine safety dataset to guide potential translational use in veterinary extrapolation.

*Neratinib* is approved at a dose of 3.4 mg/kg/day (240 mg/day) administered continuously for 12 months after completion of trastuzumab-based treatment. In the pivotal phase III ExteNET trial, this regimen was associated with a very high incidence of diarrhoea (any-grade 95%, grade 3 in 40%, grade 4 in <1%), nausea (~43%), abdominal pain (~36%), fatigue (~27%), vomiting (~26%) and decreased appetite (~12%). Side effects are alleviated by concomitant administration of antidiarrheal drugs, dose reductions (2.85 mg/kg/day (200 mg/day), 2.3 mg/kg/day (160 mg/day) and 1.7 mg/kg/day (120 mg/day)) [37,132,133].

In dogs, information about *Neratinib* is based on regulatory repeat-dose toxicology and a canine diarrhoea model study [134]. In repeat-dose studies (39 weeks) in beagle dogs, the key finding was tubular hypoplasia of the testes at doses ≥0.5 mg/kg/day [132,133]. In a dedicated beagle dog model of neratinib-induced diarrhoea, healthy female beagles received neratinib 40 mg orally once daily for 5 days, then 80 mg once daily for 23 days (28-day total), which reliably produced frequent watery stools, dehydration episodes requiring subcutaneous fluids, emesis and transient biochemical changes, but no significant histopathologic damage to the intestinal mucosa. Using this model, prophylactic crofelemer markedly reduced stool frequency and severity without requiring loperamide rescue, supporting a secretory mechanism of neratinib diarrhoea that is conserved between dogs and humans. Overall, canine data exhibit that gastrointestinal secretory toxicity appears at daily oral doses on the order of tens of milligrams per dog over weeks [134].

### 5.4. BRAF Mutation

The BRAF inhibitor *Dabrafenib* and a MEK1/2 inhibitor *Trametinib* are combined to target the MAPK pathway in tumours harbouring BRAF V600 mutations. Combine therapy recommends *Dabrafenib* at 4.3 mg/kg/day (total 300 mg/day) and *Trametinib* at 0.03 mg/kg/day (2 mg/day), continued until progression or unacceptable toxicity [135]. The combined safety profile has been characterised across large melanoma and non-small-cell lung cancer (NSCLC) trials (e.g., COMBI-d, COMBI-v, COMBI-AD). It is considered representative of BRAF V600-mutated solid tumours, including breast cancer. The most common AEs (≥20% of patients) are pyrexia, fatigue, nausea, chills, headache, diarrhoea, vomiting, arthralgia and rash [135]. Severe episodes (pyrexia (≥38 °C), rigours, dehydration, hypotension), can lead to acute kidney injury and are managed by antipyretics, dose modification (*Trametinib* 0.02 mg/kg/day (1.5 mg/day), 0.014 mg/kg/day (1 mg/day), 0.007 mg/kg/day (0.5 mg/day)) or therapy interruption [136]. Other significant combination toxicities include haemorrhagic events, cardiac dysfunction (in left-ventricular ejection fraction decrease and myocarditis), interstitial lung disease/pneumonitis, hypertension, diarrhoea, rash and ocular events (retinal pigment epithelial detachment, retinal vein occlusion) [137,138]. Overall, the combination regimen at 4.3 mg/kg/day *Dabrafenib* and 0.03 mg/kg/day *Trametinib* produces a predictable pattern of pyrexia, cutaneous toxicity, gastrointestinal, cardiovascular and ocular signals, which is manageable by dose reduction (*Dabrafenib* 2.85 mg/kg/day (200 mg/day), 2.14 mg/kg/day (150 mg/day), 1.4 mg/kg/day (50 mg/day); *Trametinib* to 0.02 mg/kg/day (1.5 mg/day), 0.014 mg/kg/day (1 mg/day), 0.007 mg/kg/day (0.5 mg/day)) or interruption [135]. In dogs, data are derived from regulatory repeat-dose toxicology studies. For *Dabrafenib*, 4-week and 13-week repeat-dose studies in beagle dogs identified the skin, heart and gonads as principal target organs. In a 4-week study (1, 5, 50 mg/kg/day), dogs developed prominent cutaneous lesions (papillomatous areas around the muzzle and chin, thickened external ear). One of six animals showed marked hypertrophy of the tricuspid valve with focal haemorrhage at 50 mg/kg/day, changes were reversible after a 2-week recovery period [139,140]. In a 13-week study (5, 20, 60 mg/kg/day (males) and 100 mg/kg/day (females)), due to severe clinical signs (inappetence, weight loss, dehydration, liquid faeces, gingival ulceration, emesis, ocular discharge), the highest doses (60–100 mg/kg/day) were stopped after 2 weeks. Dogs tolerated 20 mg/kg/day chronic dosing. Still, they showed dose-related skin papules and erosions, thymic and lymphoid depletion, lung inflammation, and marked male reproductive toxicity (testes degeneration and depletion, epididymis aspermia, glandular changes in the prostate). Cardiac lesions (hypertrophy of the atrioventricular valve and haemorrhage) occurred in some animals at ≥20 mg/kg/day [139].

For *Trametinib*, the repeat-dose dog study exposed beagle dogs to oral doses of 0.015, 0.030 and 0.060 mg/kg/day (administered initially as 0.15, 0.3 and 0.6 mg/m^2^/day), with the highest dose later reduced to 0.045 mg/kg/day, over 13 weeks [141]. The principal target organs of toxicity in dogs were the skin (lesions, scabs, preputial or vulvar swelling), gastrointestinal tract (hypersalivation), lungs (haemorrhage, mononuclear infiltration, pleural fibrosis, macrophage accumulation), and lymph nodes (sinus erythrocytosis and haemorrhage). Importantly, electrocardiographic parameters remained essentially unchanged, even at the highest tested doses, as well as in single-dose cardiovascular safety studies (doses up to 0.15 mg/kg) [137,141,142].

A dedicated combination toxicology study in dogs was conducted, administering *Dabrafenib* (0, 5, 20 mg/kg/day) together with *Trametinib* (0, 0.0075, or 0.0225 mg/kg/day). At the highest combination (20 mg/kg/day *Dabrafenib*/0.0225 mg/kg/day *Trametinib*), due to weight loss, reduced food intake, dark red/black liquid faeces and hyperthermia, one male dog was euthanised (day 11). Across dose levels, gastric inflammation with leucocytosis and neutrophilia, thymic hypocellularity, dose-dependent germ-cell degeneration/oligospermia, and testicular damage, particularly at the high-dose combination, were observed. Accumulation of trametinib at the end of the dosing period was also documented, suggesting that combination exposure may be more sustained in dogs than in humans [139].

Taken together, therapeutic exposures in humans at the combination produce pyrexia, cutaneous events, gastrointestinal, cardiovascular and ocular toxicity, which can be managed by dose reduction or interruption. In dogs, repeat-dose exposures at several-fold higher mg/kg doses of *Dabrafenib* and similar mg/kg doses of *Trametinib* result in qualitatively identical toxicities, with additional emphasis on gastric injury and male reproductive damage in the combination setting. These findings imply that if *Dabrafenib* and *Trametinib* combination were to be considered for BRAF-mutant canine tumours, dosing would need to remain well below the dog toxicology levels (e.g., ≤5 mg/kg/day *Dabrafenib* and ≤0.01–0.02 mg/kg/day *Trametinib*), with systematic monitoring of skin, gastrointestinal tract, cardiac function, reproductive organs and complete blood counts to define a safe therapeutic window in the canine species.

## 6. Conclusions

Canine mammary carcinoma remains a significant oncological challenge in companion animal medicine and an understudied comparative model for human breast cancer. Despite outstanding progress in the development of novel molecularly targeted therapies for humans, no kinase inhibitors are U.S. Food and Drug Administration (FDA)-approved for veterinary use in the treatment of canine mammary tumours. This underutilization reflects the existing gap between existing scientific knowledge and clinical practice in veterinary oncology.

Currently, standard treatment for CMC still relies almost exclusively on surgical resection of the mammary gland, that is, partial or bilateral mastectomy. However, given the complex vascular and lymphatic architecture of the mammary gland chain, the presence of numerous anastomoses, and the high metastatic potential of these tumours, surgical intervention alone rarely results in a cure, especially when the disease has already spread, as is typical in dogs. Effective treatment of advanced or disseminated disease requires systemic therapy. Traditional chemotherapy regimens, often based on platinum compounds, offer limited benefits and are associated with significant toxicity.

Over the past decade, molecular studies have significantly expanded our understanding of signalling networks and oncogenic factors in canine mammary cancer. These discoveries provide a solid scientific basis for introducing kinase inhibitors, already introduced in human breast cancer treatment, into veterinary medicine. Drugs targeting the HER2/EGFR, PI3K/AKT/mTOR, and CDK4/6-RB pathways are promising candidates. Unfortunately, this study demonstrates that only four of the twelve available drugs of this type approved by the FDA for the treatment of human mammary cancer have undergone any preclinical trials for the treatment of the same cancer in dogs. Furthermore, in all cases, these studies were conducted only in vivo on tumour cells, demonstrating that these drugs affect the proliferation and life cycle. At the same time, some of these drugs have been studied in individual clinical cases, unfortunately, on other cancers.

Importantly, for most drugs, safe doses in dogs approach the mg/kg range of therapeutic doses in humans, suggesting substantial interspecies compatibility. However, unlike in human clinical oncology, virtually no data are available on combination targeted therapies in dogs. The only exception is toxicology studies of combination therapy with MAPK inhibitors conducted for regulatory purposes. There are no systematic toxicology studies of combination therapies, nor are there clinical trials of combination therapies in dogs with spontaneous tumours, including canine mammary carcinoma. This gap severely limits the ability to predict the true tolerability of multidrug targeted therapy regimens in dogs.

To translate this acquired knowledge into clinical practice, further preclinical research is necessary. Xenograft models in mice or zebrafish could provide valuable experimental platforms for assessing efficacy, dosing, and safety before initiating studies in dogs. Ultimately, randomised clinical trials in dogs, with rigorous safety monitoring, are necessary to confirm the therapeutic value of kinase inhibitors in the treatment of CMC. This includes toxicity assessments of both single and combination therapies, as well as studies in a cohort of animals with spontaneous mammary carcinoma. Bridging this translational gap will not only improve oncological care in dogs but also deepen our understanding of the biology of mammary tumours across species.

## Figures and Tables

**Table 1:** Chronological overview of twelve key TK agents that have shaped therapeutic strategies in human oncology, with their FDA approval year, primary target(s), and current combination regimens in breast cancer management [23].

Drug	Primary Target(s)	FDA Approval (Breast Cancer)	Approved or Investigated Combination Regimens
Lapatinib	HER2/EGFR (ErbB1/ErbB2)	2007	Combined with capecitabine, letrozole, or trastuzumab for HER2^+^/HR^+^ metastatic breast cancer.
Everolimus	mTORC1	2012	Combined with exemestane in HR^+^/HER2^−^ advanced breast cancer resistant to nonsteroidal aromatase inhibitors.
Palbociclib	CDK4/6	2015	Combined with letrozole or fulvestrant in HR^+^/HER2^−^ advanced breast cancer.
Ribociclib	CDK4/6	2017	Combined with aromatase inhibitors or fulvestrant, demonstrated an overall survival benefit. (MONALEESA trials).
Abemaciclib	CDK4/6	2017	Used as monotherapy or combined with endocrine therapy; also approved for adjuvant HR^+^/HER2^−^ early breast cancer.
Neratinib	pan-HER (HER1/2/4)	2017	Extended adjuvant therapy after trastuzumab in HER2^+^ early-stage breast cancer.
Alpelisib	PI3Kα (p110α)	2019	Combined with fulvestrant in HR^+^/HER2^−^, PIK3CA-mutated advanced breast cancer (SOLAR-1 study).
Tucatinib	HER2 (selective)	2020	Combined with trastuzumab and capecitabine in metastatic HER2^+^ breast cancer, including brain metastases (HER2CLIMB).
Dabrafenib + Trametinib	BRAF V600E + MEK1/2	2022 (tissue-agnostic)	Rare BRAF-mutant breast cancers; combination approved for any solid tumour harbouring BRAF V600E.
Capivasertib	AKT1/2/3	2023	Combined with fulvestrant in HR^+^/HER2^−^ advanced breast cancer with AKT-pathway alterations (CAPItello-291 trial).
Inavolisib	PI3Kα	2024	Approved with endocrine therapy for *PIK3CA*-mutated HR^+^/HER2^−^ metastatic breast cancer; improved safety over Alpelisib.

**Table 2:** An overview of twelve key tyrosine kinase inhibitors relevant to canine mammary cancer: current evidence and knowledge gaps.

Drug	Target Pathway	Veterinary Status for CMC Treatment (Approval for Veterinary Use)	Evidence in CMC (Type)	Key Translational Comment	Source
Palbociclib	CDK4/6	Not approved	In vitro (CMT cell lines)	Activity depends on an intact RB pathway. No in vivo safety data in dogs yet.	[80]
Ribociclib	CDK4/6	Not approved	No data available	-	-
Abemaciclib	CDK4/6	Not approved	No data available	-	-
Alpelisib	PI3Kα	Not approved	In vitro (CMT organoids and cell lines)	Requires *PIK3CA* mutation. No in vivo safety data in dogs yet.	[81]
Inavolisib	PI3Kα	Not approved	No data available	-	-
Capivasertib	AKT	Not approved	No data available	-	-
Everolimus	mTOR	Not approved	In vitro (CMT cell lines)	Possible benefit in PTEN-low/PI3K-high tumours; Further preclinical validation is required	[82]
Lapatinib	HER2/EGFR	Not approved	In vitro (HER2^+^ CMT lines)	HER2 status in CMC is inconsistent. Standardised IHC/ISH testing needed	[83]
Tucatinib	HER2	Not approved	No data available	-	-
Neratinib	pan-HER	Not approved	No data available	-	-
Dabrafenib + Trametinib	BRAF V600E/MEK	Not approved	No data available	-	-

## Data Availability

These data were derived from the following resources available in the public domain: PubMed (https://pubmed.ncbi.nlm.nih.gov/ (accessed on 1 November 2025)), FDA (https://www.fda.gov (accessed on 1 November 2025)), EMA (https://www.ema.europa.eu (accessed on 1 November 2025)), Vidium Animal Health (https://vidiumah.com (accessed on 1 November 2025)).

## Abbreviations

The following abbreviations are used in this manuscript:

FDA	U.S. Food and Drug Administration
PubMed	Free full-text archive of biomedical and life sciences journal literature at the US National Institutes of Health’s National Library.
EMA	European Medicines Agency
CMC	Canine mammary carcinoma
PI3K/AKT/mTOR signalling pathway	Phosphatidylinositol 3-kinase/protein kinase B/mechanistic target of rapamycin signalling pathway
CDK4/6-RB signalling pathway	Cyclin-dependent kinases 4 and 6 pathway
RAS/MAPK signalling pathway	Mitogen-Activated Protein Kinase pathway
MAPK/ERK signalling pathway	Mitogen-Activated Protein Kinase/Extracellular Signal-Regulated Kinase pathway
RB1	Retinoblastoma protein
RTKs	Receptor Tyrosine Kinase
IGF1R	Insulin-like growth factor 1 receptor
PTEN	Phosphatase and tensin homologue
ERBB/HER family	Erythroblastic oncogene B, receptor tyrosine kinase family
HER1 (EGFR, ErbB1),	Epidermal growth factor receptor
HER2 (ErbB2)	Human epidermal growth factor receptor 2
HER3(ErbB3)	Human epidermal growth factor receptor 3
HER4(ErbB4),	Human epidermal growth factor receptor 4
ATP	Adenosine triphosphate
Da	Unit of mass (Dalton)
TK	Tyrosine kinase
PI3Kα	Phosphoinositide 3-kinase alpha
*PIK3CA*	Phosphatidylinositol 3-kinase (PI3K) gene
Pan-HER	All members of the human epidermal growth factor receptor (HER) family
HR^+^/HER2^−^	Hormone receptor-positive (HR^+^) and human epidermal growth factor receptor 2-negative (HER2^−^)
HR^+^/HER2^+^	Hormone receptor-positive (HR^+^) and human epidermal growth factor receptor 2-positive (HER2^+^)
BRAF	Proto-oncogene B-Raf and v-Raf murine sarcoma viral oncogene homologue
MEK1/2	Mitogen-activated protein kinases 1 and 2
AKT 1/2/3	Protein Kinase B (PKB) Family
ASCO/CAP	American Society of Clinical Oncology/College of American Pathologists guideline
DFG	The Asp-Phe-Gly (DFG) motif, a highly conserved sequence of three amino acids found in almost every eukaryotic protein kinase
KIT	Tyrosine-protein kinase KIT, Proto-oncogene c-KIT
VEGFR2	Vascular Endothelial Growth Factor Receptor 2
PDGFRβ	Platelet-derived growth factor receptor beta
P114	Canine mammary carcinoma cell line
CF41	Canine mammary carcinoma cell line
CTBp	Canine mammary carcinoma cell line
muPC	Canine prostatic carcinoma cell line
TCC	Transitional cell carcinoma
AE	Adverse Event
AST	Aspartate Aminotransferase
ALT	Alanine Aminotransferase
QTc	Heart-rate corrected QT interval.
ECG	Electrocardiogram
AUC	Area under the curve/Maximum tolerated exposure
LOAEL	Lowest Observed Adverse Effect Level
GLP	Good Laboratory Practice
NSCLC	Non-small-cell lung cancer
